# Real-Time and Label-Free Chemical Sensor-on-a-chip using Monolithic Si-on-BaTiO_3_ Mid-Infrared waveguides

**DOI:** 10.1038/s41598-017-05711-4

**Published:** 2017-07-19

**Authors:** Tiening Jin, Leigang Li, Bruce Zhang, Hao-Yu Greg Lin, Haiyan Wang, Pao Tai Lin

**Affiliations:** 1 Department of Electrical and Computer Engineering, Texas A&M University, College Station, Texas, 77843 United States; 2 Department of Materials Science and Engineering, Texas A&M University, College Station, Texas, 77843 United States; 3 Center for Remote Health Technologies and Systems, Texas A&M University, College Station, Texas, 77843 United States; 4 School of Materials Engineering, Purdue University, West Lafayette, Indiana, 47907 United States; 5 School of Electrical and Computer Engineering, Purdue University, West Lafayette, Indiana, 47907 United States; 6 Center for Nanoscale Systems, Cambridge, Harvard University, 11 Oxford Street, Massachusetts, 02138 United States

## Abstract

Chip-scale chemical detection is demonstrated by using mid-Infrared (mid-IR) photonic circuits consisting of amorphous silicon (a-Si) waveguides on an epitaxial barium titanate (BaTiO_3_, BTO) thin film. The highly c-axis oriented BTO film was grown by the pulsed laser deposition (PLD) method and it exhibits a broad transparent window from λ = 2.5 μm up to 7 μm. The waveguide structure was fabricated by the complementary metal–oxide–semiconductor (CMOS) process and a sharp fundamental waveguide mode has been observed. By scanning the spectrum within the characteristic absorption regime, our mid-IR waveguide successfully perform label-free monitoring of various organic solvents. The real-time heptane detection is accomplished by measuring the intensity attenuation at λ = 3.0–3.2 μm, which is associated with -CH absorption. While for methanol detection, we track the -OH absorption at λ = 2.8–2.9 μm. Our monolithic Si-on-BTO waveguides establish a new sensor platform that enables integrated photonic device for label-free chemical detection.

## Introduction

Chemical sensors using integrated photonics have attracted significant attention, due to their potential for environmental monitoring and high throughput screening for biomedical discovery^1–5^. Advanced technologies using absorbance, surface plasmon resonance (SPR), and fluorescence detection have been developed to achieve chip-scale optical sensing^6–10^. For instance, chemical sensors using optical micro-ring resonators with ppm-level detection have been demonstrated^11–14^. Meanwhile, SPR sensors using perfect absorbers or highly doped semiconductors are utilized for multispectral IR Spectroscopy and gas identification^15–18^. However, on-chip sensors capable of broadband mid-IR sensing have not yet been well developed^19, 20^. A mid-IR sensor is capable of providing label-free and real-time sensing applications because it overlaps with characteristic absorption bands of various organic and inorganic compounds^21, 22^. Prior studies utilized multilayer material platforms including Si-on-SiO_2_, Si-on-sapphire, and SiN_x_-on-SiO_2_, etc^23–28^. Though crystalline Si and amorphous SiN_x_ are transparent up to λ = 8 μm, SiO_2_ becomes opaque after λ = 3.7 µm and sapphire after λ = 4.5 µm, which exclude those platforms for mid-IR applications at longer wavelengths.

To overcome these challenges, we propose a monolithic a-Si on BTO platform for mid-IR integrated photonic devices and sensing applications because of following advantages. *i*. Both a-Si and BTO have a broad infrared transparent window up to λ = 7 μm, so a Si-on-BTO waveguide can be utilized to monitor characteristic absorption bands at longer wavelengths. *ii*. BTO has a lower refractive index **n** of 2.4 making it an ideal undercladding material for a Si waveguide with an index **n** of 3.5. The large difference of **n** between Si and the BTO ensures that the light is efficiently confined by the waveguide and consequently decreases the bending loss caused by waveguide curvatures. *iii*. Unlike other ferroelectrics, such as LiNbO_3_, BTO has the potential to be integrated on a Si wafer through various thin film deposition technologies, such as PLD, molecular beam epitaxy (MBE), and chemical vapor deposition (CVD), which enables the integration between functional oxides and Si photonics^29–33^. *iv*. BTO has high chemical stability and mechanical hardness making it capable for biochemical and toxic sensing under harsh environments. *v*. The restraint of the crystal lattice matching between the crystalline BTO layer and the upper Si device layer is relieved by the utilization of a-Si. For conventional silicon-on-insulator (SOI), it requires a crystalline Si film and involves sophisticated preparation processes, from oxygen ion beam implantation and high-temperature annealing to exfoliation. Alternatively, the proposed a-Si waveguide layer can be directly deposited and then patterned on the epitaxial BTO layer though a standard CMOS process.

In this work, we demonstrate a mid-IR Si-on-BTO waveguide for label-free chemical sensing. The waveguide structures and the waveguide mode profiles were designed and calculated by the two-dimensional finite difference method (FDM). In parallel, we prepared the epitaxial BTO thin film on LaAlO_3_ (LAO) (001) substrates by PLD and then develop the a-Si ridge waveguide on the BTO film through CMOS process. The waveguide structure and the compositions of the Si-BTO-LAO multilayer were characterized by a scanning electron microscope (SEM) equipped with energy-dispersive X-ray spectroscopy (EDX). Meanwhile, the waveguide mode profiles were recorded and analyzed at λ = 2.5 μm–3.2 μm. Selected organic solvents, heptane and methanol, were tested to evaluate the detection capability of our waveguide sensors. Chemical detection was accomplished by correlating the waveguide spectral attenuation with the characteristic absorption of the test solvents.

## Results and Discussion

**Figure 1:**
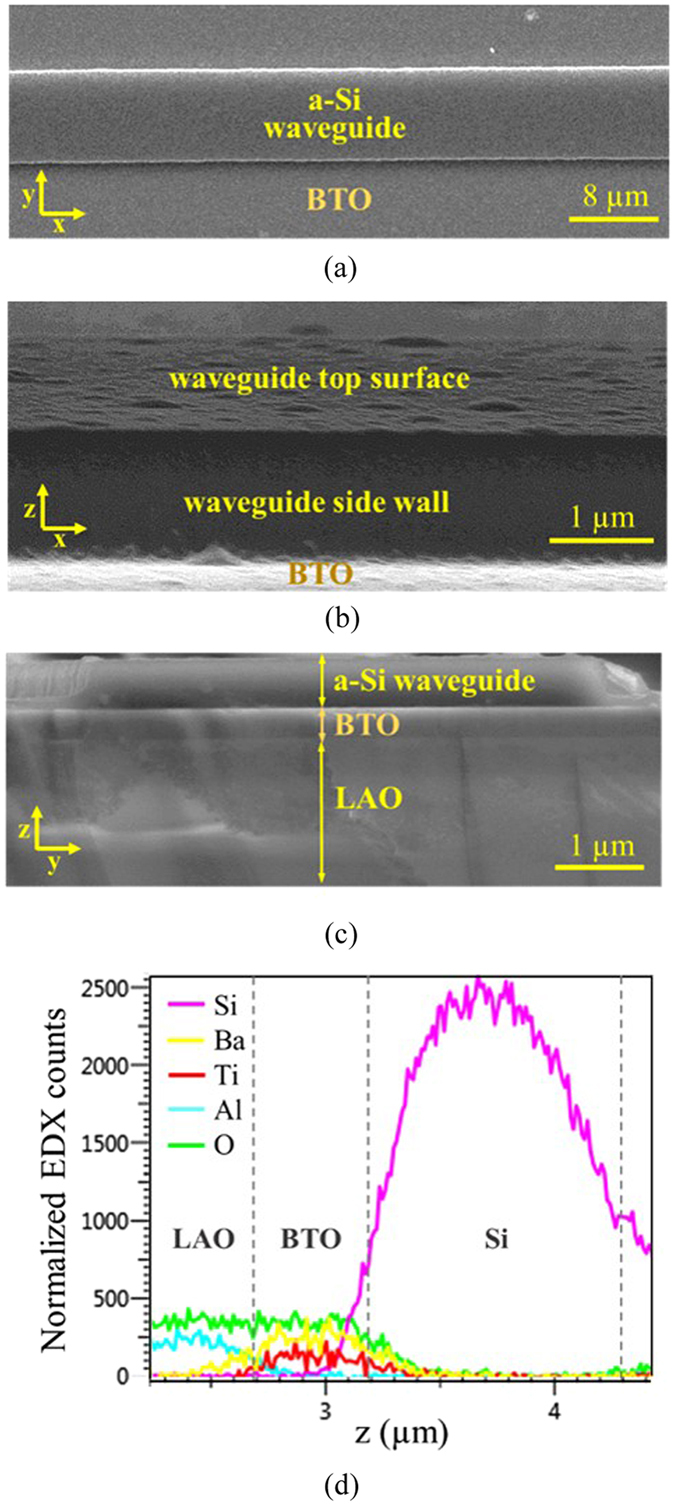
SEM images of a 10 μm wide Si-on-BTO waveguide captured from (**a**) the top, (**b**) the side, and (**c**) the cross-section of the device. A clear waveguide surface, smooth Si-BTO interface, and sharp waveguide edges were observed. (**d**) EDX element line scanning determined the structure and the composition of each device layer.

The fabricated mid-IR sensing device was inspected by a SEM with EDX. Figure 1(a) and (b) are the top and the side SEM images of a 10 μm wide a-Si on BTO waveguide. The Si layer has a well-defined ridge profile without bends or distortions found on the edge. No cracks or indents on the BTO film surface indicates that ion damage has not been introduced on the BTO film during the etching process. The sharp waveguide edges reduce the waveguide propagation loss caused by light scattering, which is critical to achieve a high signal-to-noise ratio and high sensitivity during waveguide sensing. Meanwhile, the cross-sectional image in Fig. 1(c) displays a clearly resolved interface between the top a-Si waveguide and the under-cladding BTO layer that confirms the lattice matching requirement between the Si and the crystalline BTO film relieved by using amorphous Si. The material composition of the monolithic Si-on-BTO platform was characterized by EDX using the emission lines of Si Kα at 1.74 keV and Ba Lα at 4.47 keV. The EDX scanning provides elemental distributions of the Si, Ba, Ti, and Al that are associated with the a-Si waveguide, BTO cladding layer, and LAO substrates, respectively. From the cross-sectional EDX scanning shown in Fig. 1(d), the Si waveguide height is 1 μm and the BTO film thickness is 0.5 μm, while the LAO substrate is present underneath the device layers.

**Figure 2:**
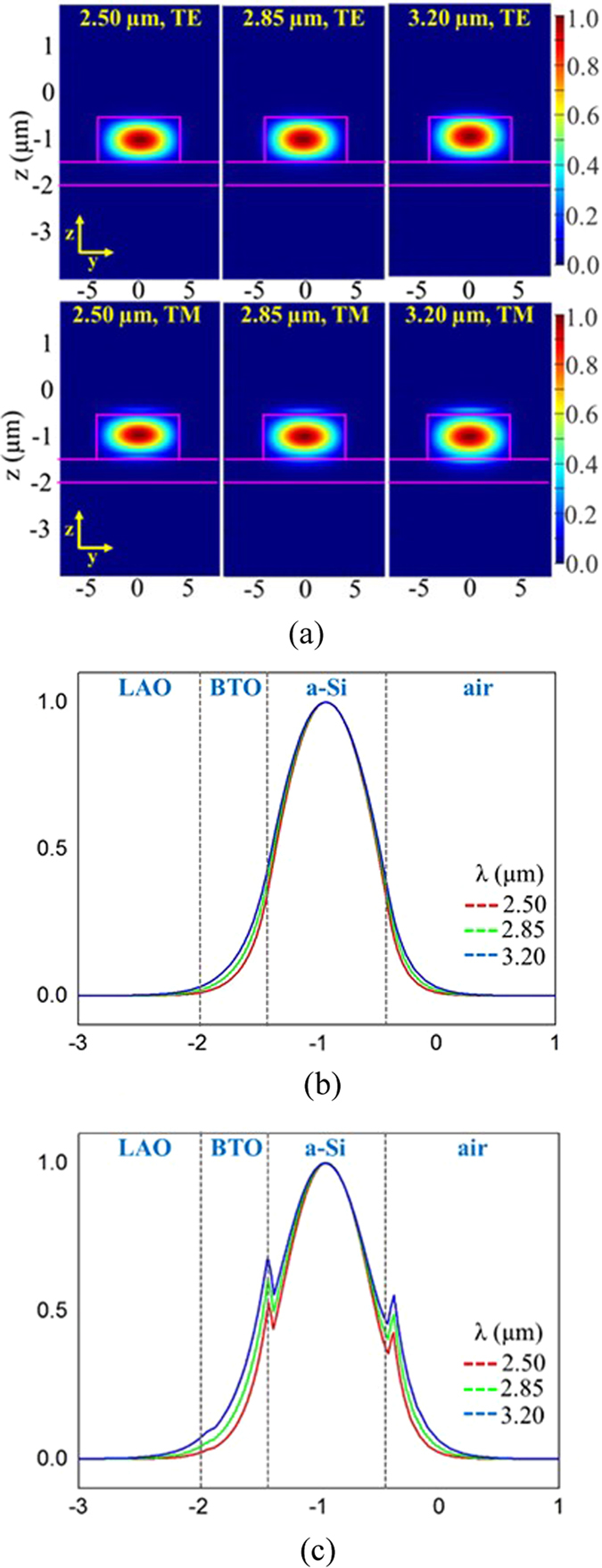
(**a**) The optical fields of the mid-IR waveguide were calculated at λ = 2.50 µm, 2.85 µm, and 3.20 µm. Fundamental modes with similar ellipsoid intensity distributions were resolved in the Si layer in all three wavelengths. (**b**) and (**c**) are the calculated 1D intensity profiles of the TE modes and the TM modes, along the z axis, respectively.

Numerical simulations of waveguide modes were calculated by using the two-dimensional finite difference method (FDM). For waveguide sensing application, it is critical to evaluate the optical fields of waveguide modes since the sensitivity of a waveguide sensor is determined by the interaction between its evanescent field and the molecules close to the waveguide surface. The structure utilized in our mode calculation was obtained from the SEM measurement, where the a-Si ridge is 1 μm tall and 10 μm wide and the BTO layer underneath the Si is 0.5 μm thick. The refractive index of a-Si, BTO and LAO were 3.5, 2.4 and 2.0, respectively. A 12 μm × 6 μm light source was chosen to excite the waveguide mode since its size is comparable to 9 μm core diameter of the mid-IR fiber used in the experiment. The intensity profiles corresponding to the TE and TM waveguide modes at λ = 2.5 μm, 2.85 μm, and 3.2 μm are depicted at Fig. 2(a). Fundamental modes with similar ellipsoid intensity distribution are clearly resolved in the Si layer over λ = 2.5 μm to λ = 3.2 μm, while the evanescent fields in the air (z > −0.5 µm) and inside the BTO layer (z < −1.5 µm) increase as the mid-IR shifts to longer wavelengths. In addition, stronger evanescent fields are found in the TM mode profile comparing to that of the TE mode. Meanwhile, both the TE and the TM modes have relatively weak evanescent fields along the y directions (y < −5 µm or y > 5 µm) because the a-Si waveguide structure has a high y/z aspect ratio of 10/1. To better analyze the mode properties, Fig. 2(b and c) display one dimensional TE and TM polarized intensity profiles along the z-axis. The relative intensities confined in each layer, air, Si, BTO, and LAO, were calculated at λ = 2.5 μm, 2.85 μm, and 3.2 μm and the results are summarized in Table 1. Both the TE and the TM modes expand their optical fields extensively into the upper air as well as the lower BTO layer. The relatively strong field found within the BTO layer is attributed to its high refractive index of **n**_**BTO**_ = 2.4. Meanwhile, the optical intensity above the waveguide is found to be stronger for the TM mode comparing to that for the TE mode. For instance, at λ = 3.2 µm the TM mode has 10.10% intensity in the air that is 1.7 times higher than 6.01% for the TE mode. In addition, the evanescent fields increase as the wavelength increases from λ = 2.5 µm to 3.2 µm. These results indicate our waveguide sensor will exhibit a higher sensitivity when it operates with TM polarization light as well as at a longer wavelength. The preservation of the fundamental mode over a wide spectral range is another necessity to achieve high accuracy of waveguide sensing. An excitation of higher order modes will alter the mode profiles and vary the intensities of evanescent fields that consequently will lead to false signals upon spectrum scanning.

**Table 1:** The intensity distribution of a Si-on-BTO waveguide, which consists of up-cladding air, a-Si waveguide, BTO undercladding, and LAO substrate.

Polarization	Wavelength (µm)	Intensity distribution in each layer (%)			
		Air	Si	BTO	LAO
TE	2.50	4.19	88.44	7.07	0.32
	2.85	5.09	85.92	8.41	0.58
	3.20	6.01	83.36	9.68	0.95
TM	2.50	6.93	81.82	10.61	0.64
	2.85	8.49	77.53	12.76	1.22
	3.20	10.10	73.10	14.77	2.07

The distributions were calculated at two polarizations, TE and TM, and three different wavelengths of λ = 2.50 µm, 2.85 µm, and 3.20 µm.

**Figure 3:**
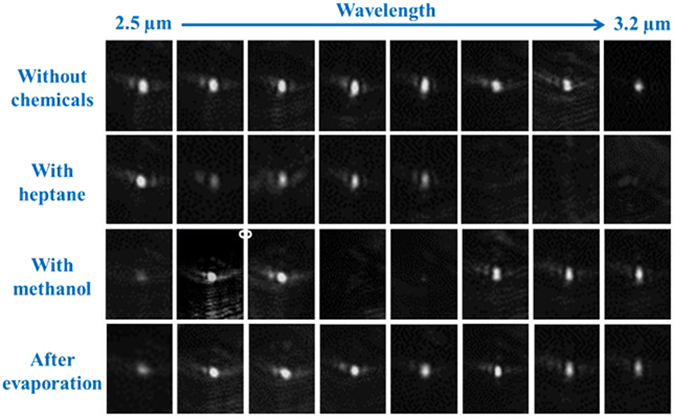
The waveguide mode images were captured from λ = 2.5 µm to 3.2 µm with or without chemicals covering the waveguide. Fundamental modes were clearly observed over a broad spectral range. When heptane was presented, the mode disappeared at λ = 3.0 µm–3.2 µm corresponding to -CH absorption. For methanol, the mode vanished at λ = 2.8 µm–2.9 µm associating with -OH absorption. The mode intensities recovered when the analytes evaporated from the waveguide surface.

In our experiments, methanol and heptane were selected as the analytes to evaluate the performance of our waveguide sensors due to their strong characterstic absorptions existing in the mid-IR regime. A light source with the TM polarization was utilized since TM light reveals a stronger evanescent field that will attribute to higher sensitivity. The wavelength of the probe light was sequentially scanned between λ = 2.5 µm and 3.2 µm because this spectrum regime overlaps with the absorption bands caused by -OH and -CH functional group while Si-on-BTO is transparent. The waveguide mode images were recorded before and after dropping the chemical analytes onto the waveguide surface. As shown in Fig. 3, without any chemicals present, a bright and sharp fundamental mode was observed through λ = 2.5 µm to 3.2 µm as expected from the simulation results. Upon dropping the heptane on the waveguide, the mode faded at λ = 3.0–3.2 µm due to the absorption caused by the -CH bond stretching. On the other hand, when methanol applies, drastic absorption appeared at λ = 2.8–2.9 µm that corresponds to the absorption due to the -OH bond stretching. Hence, our mid-IR sensor reveals distinct spectral attenuations when exposed to different chemicals, and the measured absorption results agree well with the previous studies from FTIR characterization. After the chemicals evaporate, we found that the mode profiles recovered and their intensities arrived back at the same levels just as before chemicals were applied. Thus, our mid-IR sensor is not only capable of accurate chemical identification, but also reusable.

**Figure 4:**
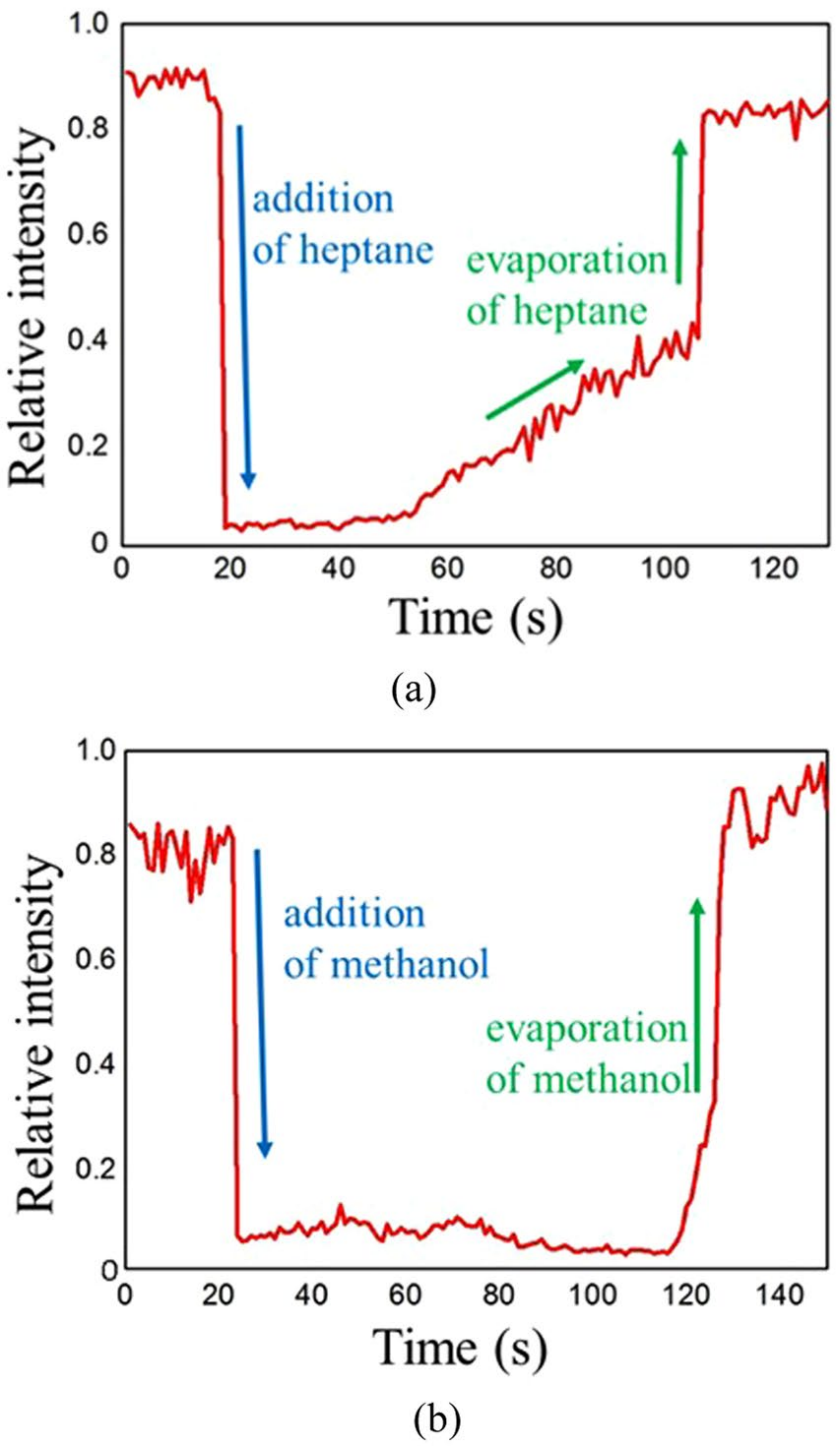
Real-time detection of (**a**) heptane and (**b**) methanol using mid-IR waveguide sensors at λ = 3.1 µm and 2.9 µm, respectively. The mode intensity decreased when the analytes were dropped on the waveguide surface and then recovered when the analyte evaporated.

The real-time chemical detection was performed by reading the transient response of the mid-IR waveguide sensors. For heptane detection, the wavelength of the probe light was tuned to λ = 3.1 µm because it is within the -CH absorption band. The waveguide mode intensity upon adding the chemical is shown in Fig. 4(a). Before t = 20 s the intensity was strong since there was no presence of any analyte. When the heptane was dropped on the waveguide surface at t = 20 s, the intensity decreased dramatically because the analyte, heptane, fully covered the mid-IR waveguide and absorbed its evanescent light. The waveguide intensity remained low until t = 50 s and then it started to recover because the heptane gradually evaporated. Eventually at t = 110 s the intensity reached its original level due to the heptane being completely left from the waveguide surface. A similar transient response was observed during the methanol sensing test shown in Fig. 4(b). To track methanol, the light wavelength was shifted to 2.9 µm to match the characteristic -OH absorption. We found that the light intensity dropped at t = 25 s, which coincided with adding methanol onto the waveguide. After a while, the intensity recovered at t = 120 s indicating the analyte evaporated from the waveguide surface. Our time-resolved characterization demonstrates that the developed mid-IR sensor is suitable for *in-situ* monitoring of various chemical analytes with high accuracy.

In summary, we demonstrated monolithic Si-on-BTO waveguides for label-free and real-time chemical detection. The epitaxial BTO thin film was prepared by pulsed laser deposition and it exhibited a broad spectral transparency from λ = 2.5 µm to 7 µm that enables our Si-on-BTO platform for mid-IR sensing application. SEM study revealed sharp waveguide side walls, as well as a smooth Si-BTO interface that reduced waveguide propagation loss, which is critical to achieve accurate chemical sensing. Heptane and methanol were then tested to exam the performance of the mid-IR waveguide sensor. Upon spectral scanning the waveguide modes showed strong intensity attenuation at λ = 3.0 µm–3.2 µm for heptane detection and λ = 2.8 µm–2.9 µm for methanol detection, corresponding to the characteristic -CH and -OH absorption bands, respectively. Furthermore, from real-time measurements, our mid-IR waveguide sensor can achieve *in-situ* chemical detection within milliseconds. The Si-on-BTO mid-IR waveguides provides a unique CMOS-compatible platform for label-free and high-throughput chemical screening.

## Methods

### Device fabrication

**Figure 5:**
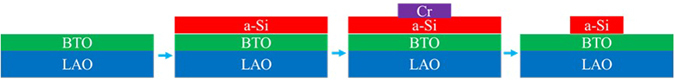
The fabrication process of the mid-IR waveguide sensor using Si-on-BTO platform. The epitaxial BTO film was grown on a (001) LAO substrate by PLD and then followed by a-Si thin film deposition through PECVD. Using photolithography and lift-off, the waveguide structure was first defined by a Cr mask, and then transferred to the a-Si layer by RIE.

Figure 5 illustrates the fabrication process of the Si-on-BTO waveguide sensor. The BTO films were deposited on single-crystal LAO (001) substrates by PLD at 10 Hz with a KrF excimer pulsed laser (Lambda Physik, λ = 248 nm). The substrate temperature was maintained at 700 °C and the O_2_ partial pressure was 40 mTorr during the deposition. After deposition, the film was annealed at 600 °C in 200 Torr O_2_ for 1 hour and then cooled down to room temperature. A 1 µm thick a-Si thin film was then deposited on the BTO film by the plasma-enhanced chemical vapor deposition (PECVD) process. The precursor gas for the a-Si deposition was SiH_4_, and the deposition temperature was 200 °C. Since the Si film is amorphous, the restraint of lattice matching between the Si film and the crystalline BTO film was relieved, which enables the formation of a smooth interface between the BTO and the Si layers. The structure of the sensor waveguide was defined through photolithography, where a 50 nm thick Cr mask was deposited on the a-Si/BTO/LAO sample by electron beam evaporation and followed by the lift-off process. The waveguide structure was then transferred to the a-Si layer by reactive ion etching (RIE). SF_6_ was used for selective Si etching, so that it did not react with the BTO film, and therefore prevented the BTO surface from becoming rough due to ion damage. It is vital to have sharp Si waveguide facets as well as a smooth BTO-Si interface to reduce the scattering loss caused by surface roughness. Previous studies that utilize HF solution for BTO etching have shown undesired rough waveguide surfaces and edges. Finally, the Cr mask and the organic residue on the device surface were removed by a ceric ammonium nitrate solution and followed by oxygen plasma ashing.

### Optical and Sensing Characterization

**Figure 6:**
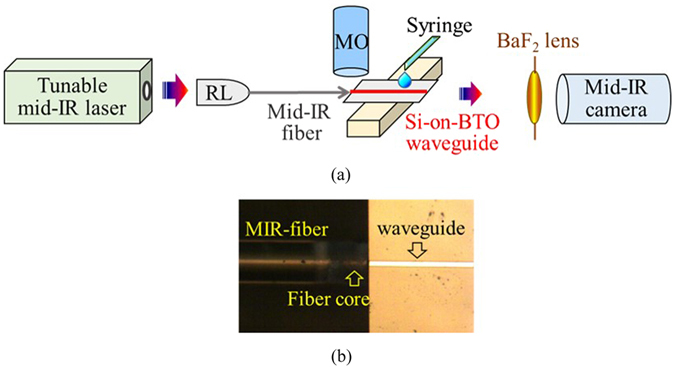
(**a**) Mid-IR test station to characterize the performance of our Si-on-BTO waveguide sensor. The probe light from a tunable pulsed laser (λ = 2.4 μm to 3.8 μm) was collimated into a mid-IR fiber using a reflective lens (RL) and then butt coupled into the waveguide. Meanwhile, the analytes was dropped onto the waveguide surface through a syringe. The mid-IR light emitted from the waveguide end facet were focused by a barium fluoride biconvex lens and then imaged by an InSb camera. (**b**) The core of the mid-IR fiber was lined up with the front facet of the Si waveguide. The fine alignment between the optical fiber and the waveguide was monitored by an upper microscope (MO).

To characterize the performance of our a-Si on BTO waveguides, a broad mid-IR test station was built and shown in Fig. 6. The light source is a pulsed laser with a wavelength tunable from λ = 2.4 μm to λ = 3.8 μm and the linewidth is of 3 cm^−1^. The laser has a pulse repetition rate of 150 kHz, a pulse duration of 10 nano seconds, and an average power of 150 mW. Using a reflective lens, the probe light was first collimated into a fluoride fiber that has a 9 μm core and 125 μm cladding, and then butt coupled into the waveguide. The core of the mid-IR fiber was lined up with the smoothly cleaved front facet of the Si-on-BTO waveguide as shown in Fig. 6(b). The fine alignment between the optical fiber and the waveguide front facet was monitored by an upper microscope equipped with a long working distance 10x objective lens. The mid-IR light emitted from the waveguide end facet was focused by a barium fluoride biconvex lens with a 25 mm focal length and then imaged by a 640 × 512 pixel InSb camera cooled by liquid nitrogen. In the chemical sensing tests, 1 mL solution was dropped from a syringe onto the Mid-IR waveguide sensor with 1 cm^2^ surface area to ensure that the solution covers the entire waveguide array. The organic solvents include heptane and methanol (≥99.9%, Sigma-Aldrich). The chemical mixtures were prepared by weight percentages. During sensing experiments the temperature was maintained at 25 °C.
